# Using data from respondent-driven sampling studies to estimate the number of people who inject drugs: Application to the Kohtla-Järve region of Estonia

**DOI:** 10.1371/journal.pone.0185711

**Published:** 2017-11-02

**Authors:** Jiacheng Wu, Forrest W. Crawford, Mait Raag, Robert Heimer, Anneli Uusküla

**Affiliations:** 1 Department of Biostatistics, University of Washington, Seattle, WA, United States of America; 2 Department of Biostatistics, Yale School of Public Health, New Haven, CT, United States of America; 3 Department of Ecology and Evolutionary Biology, Yale University, New Haven, CT, United States of America; 4 Yale School of Management, New Haven, CT, United States of America; 5 Institute of Family Medicine and Public Health, University of Tartu, Tartu, Estonia; 6 Department of Epidemiology of Microbial Disease and the Center for Interdisciplinary Research on AIDS, Yale School of Public Health, New Haven, CT, United States of America; University of Cyprus, CYPRUS

## Abstract

Estimating the size of key risk populations is essential for determining the resources needed to implement effective public health intervention programs. Several standard methods for population size estimation exist, but the statistical and practical assumptions required for their use may not be met when applied to HIV risk groups. We apply three approaches to estimate the number of people who inject drugs (PWID) in the Kohtla-Järve region of Estonia using data from a respondent-driven sampling (RDS) study: the standard “multiplier” estimate gives 654 people (95% CI 509–804), the “successive sampling” method gives estimates between 600 and 2500 people, and a network-based estimate that uses the RDS recruitment chain gives between 700 and 2800 people. We critically assess the strengths and weaknesses of these statistical approaches for estimating the size of hidden or hard-to-reach HIV risk groups.

## Introduction

Estimating the size of key HIV risk populations is difficult because these groups may be hidden, hard to reach, or socially stigmatized. People who inject drugs (PWID) often suffer from high HIV infection, but because their drug use may be criminalized, PWID may not be willing to participate in a public health research study, or to report accurately about their risk behaviors. Understanding the course of the injection drug use epidemic and reducing HIV incidence in PWID depends on accurate estimation of the number of PWID for design and implementation of harm reduction and prevention programs that reach a substantial proportion of the PWID population. Estimating the number of PWID is essential in evaluating the coverage of these programs and estimating changes in population-level characteristics such as HIV prevalence and risk behaviors.

Traditional sampling methods like capture-recapture and the multiplier method require independent random samples from the target population, which are difficult to achieve when the group of interest is hidden [1]. Capture-recapture sampling is a method for population size estimation that uses the overlap between two or more independent samples to estimate total population size, and has been widely used to estimate the incidence of disease and health-related problems [2–5]. However, most capture-recapture methods assume that the population is closed, the capture sample and recapture sample are independent, and that all individuals have the same (or known) probability of being captured. None of these assumptions can be directly tested and justified in applications of population size estimation for hidden groups [6]. The multiplier method estimates hidden population size by dividing the hidden population size with a certain trait by the proportion of the hidden population with the same trait [6]. The multiplier method may be preferable to enumeration methods when the population is difficult to reach [7]. However, the multiplier method requires a representative sample and a separate, independent data source with high quality data to yield a reasonable estimate. Since there are no standard methods to estimate the size of hidden or hard-to-reach groups for which random sampling is impossible, public health researchers sometimes combine results from multiple methods to balance the strengths and weaknesses of each method [8–12].

A newer class of methods raises the possibility that researchers can use network-structural information obtained from respondent-driven sampling (RDS), a method for recruiting research subjects through their social contacts in the target population social network [13]. RDS provides a way for researchers to quickly recruit members of a target population even when there is no readily available sampling frame. Initial subjects—called seeds—are chosen by convenience and are given a small number of coupons that they can use to recruit acquaintances who are also members of the target population. Each coupon is marked with a unique identification number. Subjects receive a reward for being interviewed and for each subject they recruit. Researchers can track who recruited whom by matching the ID of each redeemed coupon with recruiters’ ID. Each subject is interviewed and reports their network degree (i.e. egocentric network size). Recruiters cannot recruit more subjects than the number of coupons they receive, and no subject can be recruited more than once. As recruits become, in turn, recruiters, the recruitment process continues until a target sample size is reached. In order to protect privacy, subjects do not reveal identifying information about their network contacts in the hidden population. An RDS sample is not an independent random sample from the hidden population, and the traits of recruited subjects may not be independent.

RDS is typically used to estimate the average value of traits or outcomes in the population, such as HIV prevalence. Some authors have used sample averages (e.g. sample HIV prevalence) from RDS surveys as inputs to the multiplier method for estimating total population sizes [6, 14, 15, 8, 16]. Recently Handcock, Gile, and Mar [17] proposed the successive sampling (SS) method using the network degrees of recruited subject from an RDS study to estimate the size of the hidden population. Crawford, Wu, and Heimer [18] use a network-based method for population size estimation that exploits the network structure of RDS data to estimate the size of the hidden population. In this framework, the network degrees and observed pattern of recruitments impose constraints on the number of unsampled subjects connected to sampled subjects; this idea permits estimation of the total number of unsampled individuals and therefore the total size of the hidden population.

The first HIV case in Estonia was diagnosed in 1988 [19], and by 2013 the cumulative number of HIV diagnoses had reached 8702 [20]. The HIV epidemic in Estonia is mainly driven by people who inject drugs (PWID). Estonia has had the highest number of per capita drug-related fatalities in Eastern Europe; in 2012 there were 160 drug deaths in a population of 1.32 million people [21]. A recent estimate employing expert opinions placed the number of PWID in all of Estonia at approximately 9000 [22, 23]. According to the results of a capture-recapture survey, Estonia has experienced a decrease of the prevalence of injection drug use in the general population aged 15–44, from 2.7% in 2005 to 2.0% in 2008 and 0.9% in 2009 [4]. However, HIV prevalence among PWID has remained stable, slightly above 50% from 2005 to 2009 in Tallinn, Estonia, suggesting that while the absolute number of PWID may be decreasing, the burden of HIV infection in this epidemiologically important population remained high [24].

Estonian syringe exchange programs (SEPs) serving PWID were launched in 1997. Since 2007, each participating injection drug user has received an average of 117 sterile syringes per year [25, 26]. Researchers have emphasized the importance of HIV prevention programs targeting new injectors [27], and findings suggest that HIV incidence among recently initiating injectors has decreased since implementation of large-scale SEPs [24]. Within Estonia, injection drug use and HIV prevalence are especially high in the Ida-Viru county region of Kohtla-Järve, the fifth-largest city in Estonia. An RDS study in 2012 among injecting drug users found that HIV prevalence among 600 PWID in Kohtla-Järve region (the city of Kohtla-Järve and Jõhvi parish) was 61.8% [28]. However, there are currently no population size estimates for PWID in Kohtla-Järve region, so it remains uncertain whether SEPs meet existing need for clean needles.

In this paper, we evaluate three approaches to estimate the number of PWID in Kohtla-Järve region, Estonia using data from an RDS study of 600 PWID conducted in 2012. To estimate the size of this population, we employ three complementary statistical approaches, each relying on different assumptions. The first is the standard multiplier method where the number of PWID among antiretroviral treatment (ART) patients is divided by an estimate of the proportion of PWID who receive ART. The second approach is the SS method, which uses the ordered sequence of recruited subjects’ network degrees. The third method is the network-based method, which uses network-structural information from the RDS recruitment process. For this approach, we report point estimates under an idealized recruitment model and semi-parametric bounds that do not rely on strong assumptions about the recruitment process. Results from these three statistical approaches exhibit reasonable agreement. We discuss the significance of these findings in the context of the HIV epidemic in Estonia and assess the possibilities and limitations of using RDS data to estimate the size of hidden and hard-to-reach populations.

## Methods and results

### RDS data

The 2012 RDS study of PWID in the Kohtla-Järve region (city of Kohtla-Järve and Jõhvi parish), Estonia, was carried out from May to July [29]. Participants who were more than 18 years old, reported injecting drugs in the past four weeks, and spoke Russian or Estonian were eligible to participate in the study. Written informed consent was obtained from all subjects. The study protocol, including written consent procedures, was approved by both the Research Ethics Committee of the University of Tartu and the Yale University Institutional Review Board. Participants were also tested for HIV and received post-testing counseling including referral to HIV and substance abuse treatment facilities as needed.

**Fig 1:**
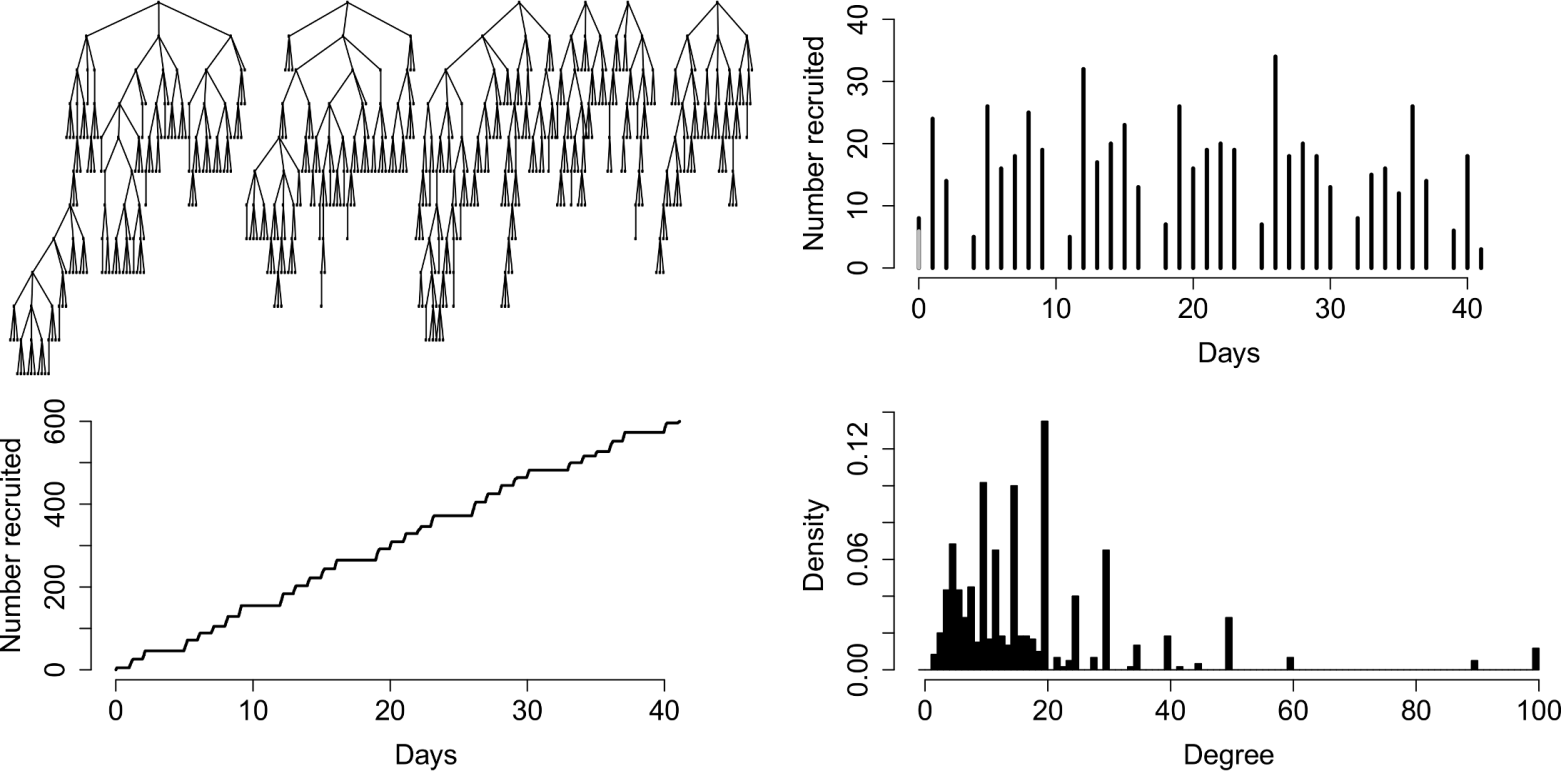
Descriptive summary of RDS data of PWID in Estonia in 2012. The top left panel shows the recruitment tree of 600 recruited subjects originating from six seeds. The top right panel shows the number of recruited subjects daily; gaps correspond to weekends. The bottom left panel illustrates the cumulative number of recruitment at each day of the study. The bottom right panel shows a histogram of subjects’ reported degrees.

In this study, *n* = 600 eligible subjects were recruited from six seeds. Each subject was given three coupons to recruit others. Fig 1 gives a descriptive summary of the RDS recruitment. The top left panel shows recruitment trees of subjects originating from six seeds. The top right panel illustrates the number of recruited subjects per day, with gaps corresponding to breaks in recruitment on weekends. The bottom left panel shows the cumulative number of recruitments at each day until the end of the study. The bottom right panel shows a histogram of the reported degrees of sampled subjects. Average degree is 17.6 (SD 15.1). The average number of recruits per day is 20. The average time for a recruiter to recruit another subject is 5.69 (SD 6.23) days.

### Multiplier method

We first present the analysis from the multiplier method since it is a standard approach for population size estimation. The method requires two pieces of information: the number of people *M* with a certain trait in the population, and the proportion *P* of people with the trait. The object of interest is *N*, the population size. The estimation formula is $\hat{N}=M/P$, where $\hat{N}$ is the estimated population size.

We choose antiretroviral treatment (ART) as the trait and estimate the number of PWID among ART patients in Kohtla-Järve region. In Estonia, ART is available free of charge in five major hospitals [30]. We assume that all ART patients in Kohtla-Järve region go to Ida-Viru central hospital for ART since previous studies report that the number of Kohtla-Järve region resident receiving ART in other hospitals is very small [31]. There were 605 patients receiving ART care in Ida-Viru central hospital in March 2013, and we use this number to estimate the number of ART patients in 2012.

While the exact number of PWID receiving ART in Ida-Viru central hospital is not readily available, we can derive an approximation based on simple random sampling to estimate it. In a recent study of ART adherence, 318 patients were recruited. Among these 318 patients, 57 to 58 subjects who were residents in Kohtla-Järve region reported receiving ART currently and injecting drugs during last 4 weeks. Not all subjects in the ART adherence study were living in the Kohtla-Järve region. If we include neighboring municipalities, the number of PWID in ART is 57. If we include all the municipalities in Ida-Viru county, the number of PWID in ART is 58. Because we have no reason to prefer one of these values over the other, we took the empirical average, and hence we use 57.5 for the calculation. Using the finite population correction for variance of the sample mean, we estimate the number of PWID among ART patients in Kohtla-Järve region as 109 people (95% CI 91–128).

Next, we use results from the RDS study to estimate the proportion of PWID who receive ART treatment. Among 600 participants, one patient filled out the questionnaire partially and 12 patients who reported being HIV+ had negative HIV test results; we assume that these people are not currently on ART in Kohtla-Järve region. Of the 600 subjects, 123 reported being currently on ART in Kohtla-Järve region. The raw proportion of PWID currently on ART in Kohtla-Järve region among 600 subjects is 20.5% (95% CI 17.27%–23.73%). The RDS Analyst software was also used to weight estimators according to their network degree, and the RDS sequential sampling estimate for the proportion is 16.66% (95% CI 12.74%–19.27%).

The estimated number of PWID is the ratio of the number of PWID receiving ART and the proportion of PWID on ART. We use the delta method to calculate the variance of the ratio and form a confidence interval. The total number of PWID in Kohtla-Järve region using the raw (unweighted) proportion is estimated to be 532 people (95% CI 413–654), and the estimated number of PWID using the weighted proportion is 654 people (95% CI 509–804).

### Successive sampling method

The SS method estimates population size from a different perspective: it only requires the ordered sequence of network degrees and does not utilize information about network structure in the RDS recruitment chain [17]. The method assumes that subjects with higher degree are more likely to be sampled earlier in the recruitment process; under this model the rate of decrease in the average network degree of sampled subjects provides information about the size of the hidden population [32, 17]. Table 1 presents the results from the SS method implemented in the “sspse” package for R [33]. Imputation of degree is a technique for smoothing the degree distribution employed by the “sspse” package. The Beta distribution is used as a prior for the sample fraction, and hence total population size, and the uniform prior with maximum possible population size 1500 and 2500 is used as a prior over the total population size. Posterior means, 95% quantiles and implied prevalence of injection drug use (assuming total population size of 44,721 people [34]) are reported.

**Table 1:** Estimates from the SS method of the number of people who inject drugs in the Kohtla-Järve region, Estonia. We obtain posterior estimates under two degree conditions (imputed and raw degree) and two priors for population size (beta and uniform). Imputed degree substitutes the raw degree with the fitted degree by Conway-Maxwell-Poisson distribution. The beta prior models the proportion of sampled subjects among target population. We set the maximum possible number of population size as 1500 and 2500 in uniform prior. Posterior mean, 5% and 95% quantiles are reported.

Degree	Prior size	Posterior Mean	95% Posterior Quantile	Implied Prevalence
Imputed	Beta	801	(621,1106)	1.8%
Imputed	Uniform[0,1500]	1104	(686,1463)	2.5%
Imputed	Uniform[0,2500]	1546	(739,2399)	3.5%
Raw	Beta	918	(600,2002)	2.1%
Raw	Uniform[0,1500]	1107	(600,1497)	2.5%
Raw	Uniform[0,2500]	1320	(600,2489)	3.0%

The SS method assumes that the average degree of recruited subjects decreases over the course of the study [17]. We evaluated this assumption by conducting a regression analysis for the slope of time-ordered network degrees [35]. We fit linear, Poisson, and M-estimates with Huber and bisquare weighting. Results are given in S1 File. We find that the slope is negative for all these methods, so we conclude that the assumption of decreasing degrees appears to hold in this study.

### Network-based method

Social or drug use connections between PWID in Kohtla-Järve region form a network where the degree of each subject is the number of PWID they know. Under statistical assumptions about homogeneity of link probabilities in the population social network, Crawford et al. [18] show that the observed data in RDS can provide information about the number of unsampled subjects connected to sampled subjects, and by extension the target population size.

We employ a standard vague prior for the population size, *π*(*N*) ∝ *N*^−1^. A Beta prior distribution *Beta*(*α*,*β*) is used for the density of connection probability *p* in the population network. S1 File details the procedure used to specify the values of the prior parameters *α* and E[p]. Table 2 shows point estimates and upper and lower bounds of the number of PWID under different prior specifications. The posterior mean E[N|Y], 95% posterior quantiles, and implied prevalence of injecting drugs in the Kohtla-Järve region are reported. Point estimates range from 1,911 people to 2,218 people. The 95% posterior quantile of lower and upper bounds are also reported. Smaller values of E[p] lead to larger point estimates and bound estimates. The lower bound of 700 reflects the minimum number of unique PWID implied by links revealed by the RDS sample and subjects’ reported degrees (see S1 File for a more detailed explanation). While the semi-parametric bounds are wider than the posterior quantiles around the point estimates, they may be more credible because they rely on fewer assumptions about the recruitment process.

**Table 2:** Estimates of the number of PWID in the Kohtla-Järve region from the network-based method. The first two columns are prior mean E[p] of the link probability, and *α* is a scale parameter for the prior. Columns 3 to 5 shows the result of point estimates with posterior mean, 95% posterior quantiles and implied prevalence of injection drug use among all 44,721 people in Kohtla-Järve region. The last column shows semi-parametric bounds for the population size.

Prior Parameter		Point Estimate			Bound Estimates
E[p]	*α*	E[N|Y]	95% Posterior Quantile	Implied Prevalence	Posterior Quantile of Lower and Upper Bound
0.00393	3	2202	(1851, 2713)	4.9%	(700, 2805)
	5	2218	(1866, 2739)	5.0%	(700, 2920)
0.01617	3	2089	(1791, 2504)	4.7%	(700, 2635)
	5	2027	(1738, 2419)	4.5%	(700, 2631)
0.02841	3	2016	(1716, 2439)	4.5%	(700, 2581)
	5	1937	(1679, 2286)	4.3%	(700, 2589)
0.04065	3	2048	(1733, 2466)	4.6%	(700, 2644)
	5	1911	(1655, 2270)	4.3%	(700, 2552)

**Fig 2:**
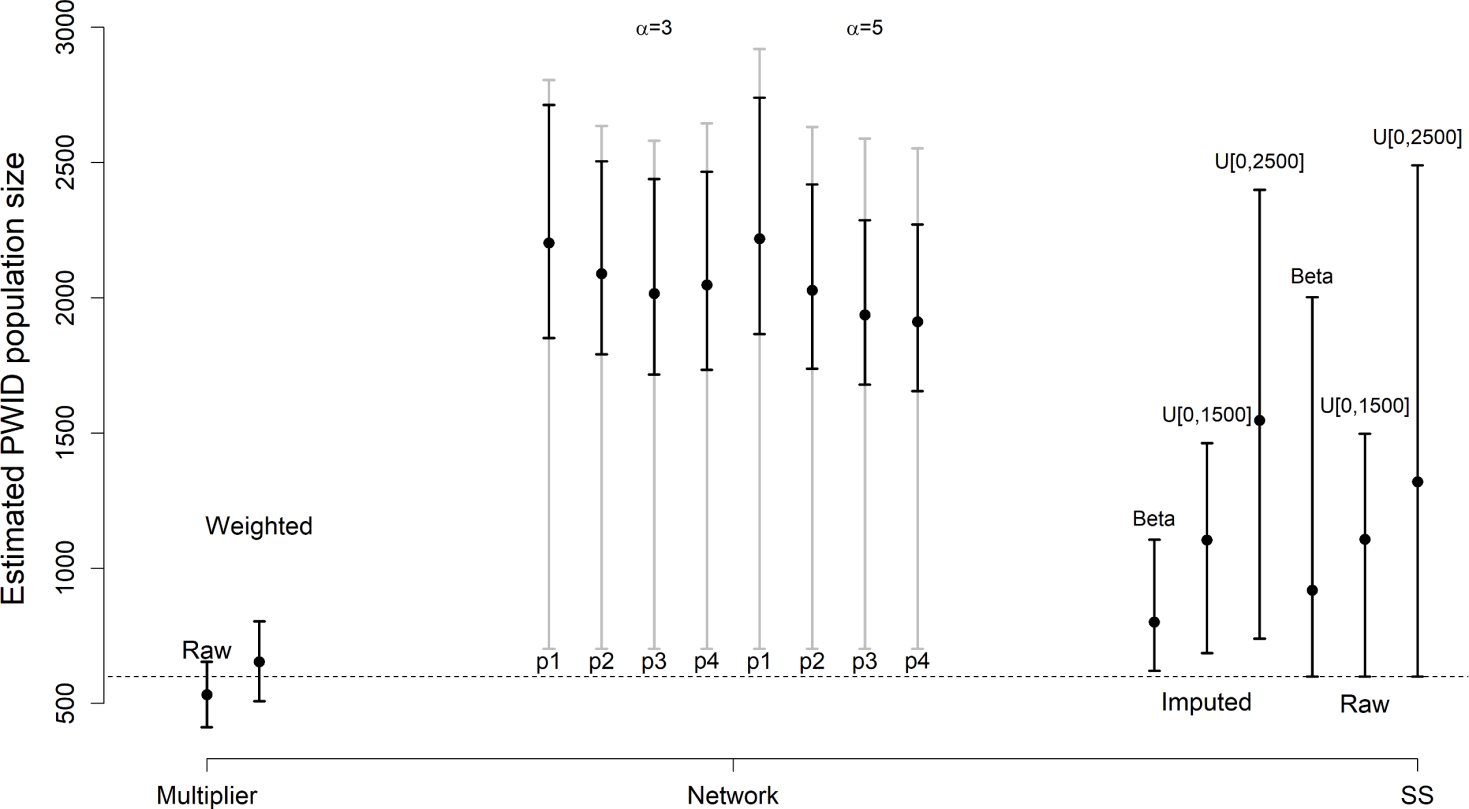
Comparison of results from the multiplier, network-based, and SS methods for population size estimation. The dashed horizontal line is the minimum number of population size (600); this is a lower bound for the PWID population size. For the multiplier method, results from the raw and weighted proportion of traits are presented. For the network-based method, results from Table 2 are shown, where the prior mean of the link probability and *α* vary. Black points and lines correspond to point estimates and posterior quantiles while grey lines represent the semi-parametric bounds. For the SS method, results from Table 1 are shown where prior selection and degree specification vary.

Fig 2 provides a visual comparison of the results from three methods. The weighted and unweighted estimates from the multiplier method exhibit some overlap, but the upper confidence limit for the weighted estimate is much larger. Importantly, the point estimate from the unweighted multiplier estimate and the lower confidence limits from both the unweighted and weighted multiplier estimates fall below 600 (shown as a dashed line), which is the number of unique PWID who participated the RDS study. The network-based method produces tighter posterior density intervals (black lines) around the point estimates, but these estimates are sensitive to the specified prior parameters. Semi-parametric bounds from the network-based method (gray lines) are much wider with lower bound close to 700. Although these semi-parametric bounds are wider than posterior quantiles around the point estimates, they may be more credible because they rely on less restrictive assumptions about the recruitment process. The point estimates and posterior intervals of SS method depend more heavily on prior specification of the maximum population size, here either 1500 or 2500 people. Using subjects’ raw reported degrees in the SS method gives wider posterior quantiles than using imputed degrees for the beta prior.

## Discussion

In this paper, we compare the multiplier method, network-based method, and the SS method for estimating the size of PWID in the Kohtla-Järve region (city of Kohtla-Järve and Jõhvi parish), Estonia. The multiplier method is well-established in public health research, and has been used in published estimates of the number of PWID in different contexts and regions [6, 14, 15, 8, 16]. The RDS-based methods of Handcock et al. [32] and Crawford et al. [18] are newer and are not currently in wide use. Each of these methods relies on different assumptions that may not be met in practice. Though the three methods are quite different in their assumptions and modes of inference, Fig 2 shows some agreement in estimates; in particular, the SS and network-based methods exhibit overlapping posterior bounds for the population size. Below we outline specific limitations and strengths of each method in the context of PWID population size estimation in Kohtla-Järve region.

Our application of the multiplier method is subject to several limitations. First, patients may have received ART from other hospitals besides Ida-Viru central hospital, which could lead to under-estimation of the numerator *M* and hence under-estimation of the population size. Second, the exact number of patients receiving ART in 2012 is not available, so we use ART data from March 2013 to approximate this number; if the number or proportion of PWID on ART changed dramatically during 2012–2013, the multiplier estimates could be subject to error. In August 2013 there were 605 ART receivers in Ida-Viru central hospital; in August 2011 there were 415 ART receivers in Ida-Viru central hospital. So the number for August 2012 may lie between those numbers. Third, the total number of people who receive ART and inject drugs is not directly observed, so we estimate this number. This estimation may not be accurate since it is possible that ART patients may conceal their drug use, possibly leading to underestimation of the number of PWID on ART and hence the total number of PWID. Fourth, it is possible that not all patients receiving ART at Ida-Viru hosptial resided in the Kohtla-Järve region. But based on the National Institute for Health Development report [31], at least 86% of the area served by Ida-Viru hospital coincides with the RDS study catchment area. Overestimation may occur if ART patients outside Kohtla-Järve region are included in our analysis. Slight changes in the demographic and geographic definitions of hidden population from different data sources can lead to differing results and may result in bias [1]. Fifth, the multiplier method assumes that RDS delivers a random sample of PWID. If the sampled HIV+ PWID were more (or less) likely to receive ART, then multiplier estimates may be in error.

The network-based and SS methods rely on data gathered by RDS, which typically are used to estimate population-level characteristics of certain traits, such as HIV prevalence. However, Handcock et al. [32] and Crawford et al. [18] suggest that data collected in the course of an RDS study may also provide useful information about the size of the target population.

The SS method also has several limitations. First, it assumes that at each step in the recruitment process, the new recruit is drawn at random from all yet-unrecruited members of the target population, with probability proportional to their network degree. This assumption ignores the relationship between the underlying social network (on whose links the RDS recruitment process is supposed to operate) and the chain of recruitments. However, this sampling assumption may be warranted when recruitment happens independently of a network. For example, if new recruits are chosen by recruiters according to their “popularity” in the target population, and reported network degree is a surrogate measure of popularity, the assumption may hold. Second, the assumption that subjects with higher degree are more likely to be sampled (i.e. receiving a coupon and successfully redeeming it) earlier in RDS may not hold in practice. Third, like the network-based method, the SS estimate is sensitive to user-specified prior information about population size and the population degree distribution. In particular, estimates from the SS method appear to be sensitive to the maximum *a priori* limit on the population size.

The network-based method employs assumptions about the recruitment process and the population social network to estimate the hidden population size. The point estimates reported in Table 2 rely on a parametric model for recruitment waiting times. While the semi-parametric bounds are less precise, they may be more credible in the sense that the true value of the PWID population size is more likely to lie within the bounds. Like the multiplier and SS methods, the network-based method has several limitations. First, it assumes equal and independent probability of two individuals sharing a social link in the underlying population network. This assumption reflects researchers’ *a priori* limited knowledge about the social structure of hidden population. This homogeneity assumption is essentially the same as that employed by capture-recapture [15] and network scale-up methods [36] to make inference from sample to population. However, the assumption may not be valid in highly clustered networks, where the probabilities of network ties are more heterogeneous [37, 38]. Second, since researchers must specify prior distributions for unknown parameters, hidden population size estimates are sensitive to prior beliefs about the network and recruitment process. Prior distributions that assign large weight to low connection probability lead to larger population size estimates and bound estimates for population size. Another limitation of the network-based method is that it requires good record-keeping of recruitment information from the RDS sample. In the data we analyze here, there were discrepancies in subject IDs due to data entry errors during recruitment that had to be rectified before analysis could proceed. It is possible that errors in recorded recruitment information could result in biased estimates of population size.

## Conclusion

In this paper, we have used data from a large RDS study to estimate the number of PWID in the Kohtla-Järve region using the multiplier method, the network-based method, and the SS method. The credibility of the assumptions underlying each estimate must be critically assessed by researchers who use RDS to estimate population size. We are hopeful that these techniques, along with future refinements, can assist public health researchers and policymakers in obtaining information vital in determining the proper scale of effective education, treatment, and intervention campaigns for PWID and other risk populations.

Evidence supports the effectiveness of harm reduction programs such as syringe exchange programs in preventing the transmission of HIV among PWID, but PWID may not benefit when programs are not scaled to the size of the at-risk population [39]. For example, a syringe exchange program based on the multiplier estimates from this paper might be unable to meet the actual need for clean needles. One reason for seeking an accurate estimate of the number of PWID in Kohtla-Järve region is to better define this epidemiologically important population reducing uncertainty about the scale of the drug abuse epidemic. More generally armed with information about the number of PWID in a region, public health researchers and policymakers may determine that there is a need to increase program attendance and then better tailor their efforts to reduce stigma and seek public support for health-related initiatives.

## Supporting information

S1 File Appendix. Data description, statistical details, and comparison to the SS method.
(DOCX)

S1 FigUnknown degree.Illustration of $d_{i}^{u}$, the number of unrecruited subjects connected to subject i at the moment when i is recruited.(TIF)

S2 Fig Time-ordered degrees. Linear regression on time-ordered network degree.
(TIF)

S1 Dataset Respondent-driven sampling data. Subject ID, recruiter ID, date of recruitment, network degree, number of coupons, and ART status for each subject.
(CSV)
