# Supplementary material for: Using data from respondent-driven sampling studies to estimate the number of people who inject drugs: Application to the Kohtla-Järve region of Estonia
Source: PLoS One. 2017 Nov 2;12(11):e0185711. doi: 10.1371/journal.pone.0185711 (PMC5667832; doi:10.1371/journal.pone.0185711)
Supplement: S1 File — Data description, statistical details, and comparison to the SS method. (DOCX) [file pone.0185711.s001.docx]

# Appendix

## Data

This study includes 600 subjects recruited by RDS, originating from six seeds. Each subject was given three coupons to recruit others. All 600 subjects are included in multiplier, network-based and SS method. The Kohtla-Järve region RDS study recruitment area includes Kohtla-Järve city and Jõhvi parish (including Jõhvi city) with a population of 44,721 in 2012 among which 33,146 were from Kohtla-Järve city and 11,575 from Jõhvi parish (including Jõhvi city). Data is in the file “estonia.csv”.

The original data set contained errors and discrepancies that were corrected manually. Seventeen subjects reported the network degree as 0 or missing; these were replaced by the subject’s implied degree in the recruitment chain. Two subjects were listed as being recruited before the study began due to a data entry error. One subject’s time of recruitment was missing; we replaced this value with the average of the time between the time when that subjects’ recruiter entered the study, and when the subject’s recruits entered the study. Thirteen subjects’ records had an incorrect coupon number; cross-checking recruiters’ coupon IDs resolved the issues.

## Network-based method

### Inference for population size

We detail the network-based method of population size estimation in this section. PWID in Kohtla-Järve region and their social connections form a network where the degree of each subject is the number of adult PWID they know. The observed data $Y$ from RDS is composed of subjects' IDs and recruiters' IDs, subjects' network degrees, and times of recruitment. The recruitment chain obtained from the observed data represents a partial observation of the subnetwork of respondents. We assume that each subject has a constant probability $p$ of being linked to any other person in the PWID population. Let $d_{i}^{u}$ be the number of unrecruited subjects that are connected to subject $i$ at the moment when $i$ is recruited (indices $i$ are ordered by the time of entry into the study). S1 Fig illustrates the meaning of$d_{i}^{u}$. At the moment that subject $i$ is recruited there are $N-i$ subjects who are still not recruited and their probabilities of being connected with subject $i$ are the same $p$. Then we have

$$d_{i}^{u}\sim Binomial\left( N-i,p \right).$$

Therefore $d_{i}^{u}$ contains information about the number of unsampled subjects, and can be used to derive the likelihood of $N,p$. Since the subnetwork of recruited subjects is not directly observed in an RDS study, $d_{i}^{u}$ is unknown. The likelihood of $N,p$ is

$$L\left( N,p | d_{i}^{u} \right)=\left[ \prod_{i=1}^{n} \left( \begin{aligned} N-i \\ d_{i}^{u} \end{aligned} \right) \right]p^{d_{i}^{u}}(1-p)^{N-i-d_{i}^{u}}.$$

### Posterior distribution of $\boldsymbol{N}$

A Bayesian procedure is utilized to calculate the posterior distribution of the target population size, obtained by multiplying the likelihood by the prior information of population size $N$ and of probability of connection $p$ based on Bayes Theorem. We assume a beta prior $Beta(\alpha,\beta)$ for the connection probability $p$ in the network, and $\pi\left( N \right)$ is the prior for the size of the target population. The posterior distribution of $N$ is proportional to

$$f\left( N | d^{u} \right)\propto\left[ \prod_{i=1}^{n} \left( \begin{aligned} N-i \\ d_{i}^{u} \end{aligned} \right) \right]B(D^{u}+\alpha,nN-\left( \begin{aligned} n+1 \\ 2 \end{aligned} \right)-D^{u}+\beta)\pi(N)$$

where $B\left( \cdot,\cdot\right)$ is the Beta function and $D^{u}=\sum_{i=1}^{n} d_{i}^{u}$.

The posterior distribution does not have closed form, so a Metropolis-Hasting algorithm is used to generate Markov Chain whose stationary distribution is the posterior distribution of $N$.

### Semi-parametric bound estimates of population size

For the lower bound let $r_{i}$ be the total number of subjects recruited by subject $i$. The number of unsampled subjects connected to subject $i$ at the moment of its recruitment$d_{i}^{u}$ cannot be smaller than $r_{i}$. Also, there are at most $i-1$ connections with subject $i$ before its recruitment thus the lower bound is

$$d_{i}^{u}\geq\max\left\{ r_{i},d_{i}-i+1 \right\}$$

For the upper bound, $d_{i}^{u}$ cannot be greater than the total degree $d_{i}$ if $i$ is seed or $d_{i}-1$ if $i$ is not seed. Let $M$ be the set of seeds. The upper bound for $d_{i}^{u}$ is

$$d_{i}^{u}\leq d_{i}-1\left\{ i\notin M \right\}$$

Let $d_{i}^{lo}=\max\left\{ r_{i},d_{i}-i+1 \right\}$ and $d_{i}^{hi}=d_{i}-1\left\{ i\notin M \right\},$then $d_{i}^{lo}\leq d_{i}^{u}\leq d_{i}^{hi}$. Substitutions of $d_{i}^{u}$ with its lower bound $d_{i}^{lo}$and upper bound $d_{i}^{hi}$ respectively lead to the posterior distribution of the lower and upper bound of PWID size.

### Point estimate of population size

The second approach is to average all potential values of $d_{i}^{u}$ by marginalizing over all subnetworks of recruited subjects that are compatible with the observed RDS data. An exponential waiting time model is used to express the joint likelihood of the network and the waiting time rate; the details are outlined in [20].

### Prior selection

We consider uninformative power law-prior distribution $\pi(N)\propto N^{-1}$ for the population size $N$. The prior for $p$ can be determined empirically. One simple estimate for $p$ depending on $d_{i}^{u}$ and a prior population size estimate $\hat{N}$ is

$$\hat{p}=\frac{\sum_{i=1}^{n} d_{i}^{u}}{n\hat{N}-\left( \begin{aligned} n+1 \\ 2 \end{aligned} \right)}$$

Although $d_{i}^{u}$ is not known *a priori*, we can bound this probability by

$$\frac{\sum_{i=1}^{n} max\left\{ r_{i},d_{i}-i+1 \right\}}{n\hat{N}-\left( \begin{aligned} n+1 \\ 2 \end{aligned} \right)}\leq\tilde{p}\leq\frac{\sum_{i=1}^{n} d_{i}-1\left\{ i\notin M \right\}}{n\hat{N}-\left( \begin{aligned} n+1 \\ 2 \end{aligned} \right)}.$$

Since the sample size of RDS is 600, we know with certainty that there are at least 600 PWID. To specify$\hat{N}$ we consider the conditional mean of population size from multiplier method as our prior inputs to network and SS method. We use normal approximation to the numerator and denominator in the multiplier method to calculate the mean of the ratio, conditional on at least 600 people using simulations. The conditional mean of the number of PWID from multiplier method is 709, so this serves as the prior estimate $\hat{N}$ above. Let $p_{lo}$ and $p_{hi}$ be the lower and upper bound respectively. For any value $\tilde{p}$ in $[p_{lo},p_{hi}]$ we can find $\beta$ using the formula $\beta=\alpha(1-\tilde{p})\tilde{p}$.

## SS method: regression on time-ordered network degree

**S1 Table. Regression analysis on time-ordered network degree.** We fit linear, Poisson, M estimates with Huber and bisquare weighting, and we find that the slope is negative for all these methods, indicating that degrees appear to decrease in this RDS dataset.

| **Method** | **Slope** | **SE** | **p-value** |
| --- | --- | --- | --- |
| Linear | -0.012 | 0.0035 | 0.0006 |
| Poisson | -6.91E-4 | 5.64E-5 | <2E-16 |
| M (Huber) | -0.0078 | 0.0023 |  |
| M (Bisquare) | -0.0058 | 0.0022 |  |
