# Supplementary figures and images for: Using data from respondent-driven sampling studies to estimate the number of people who inject drugs: Application to the Kohtla-Järve region of Estonia
Source: PLoS One. 2017 Nov 2;12(11):e0185711. doi: 10.1371/journal.pone.0185711 (PMC5667832; doi:10.1371/journal.pone.0185711)

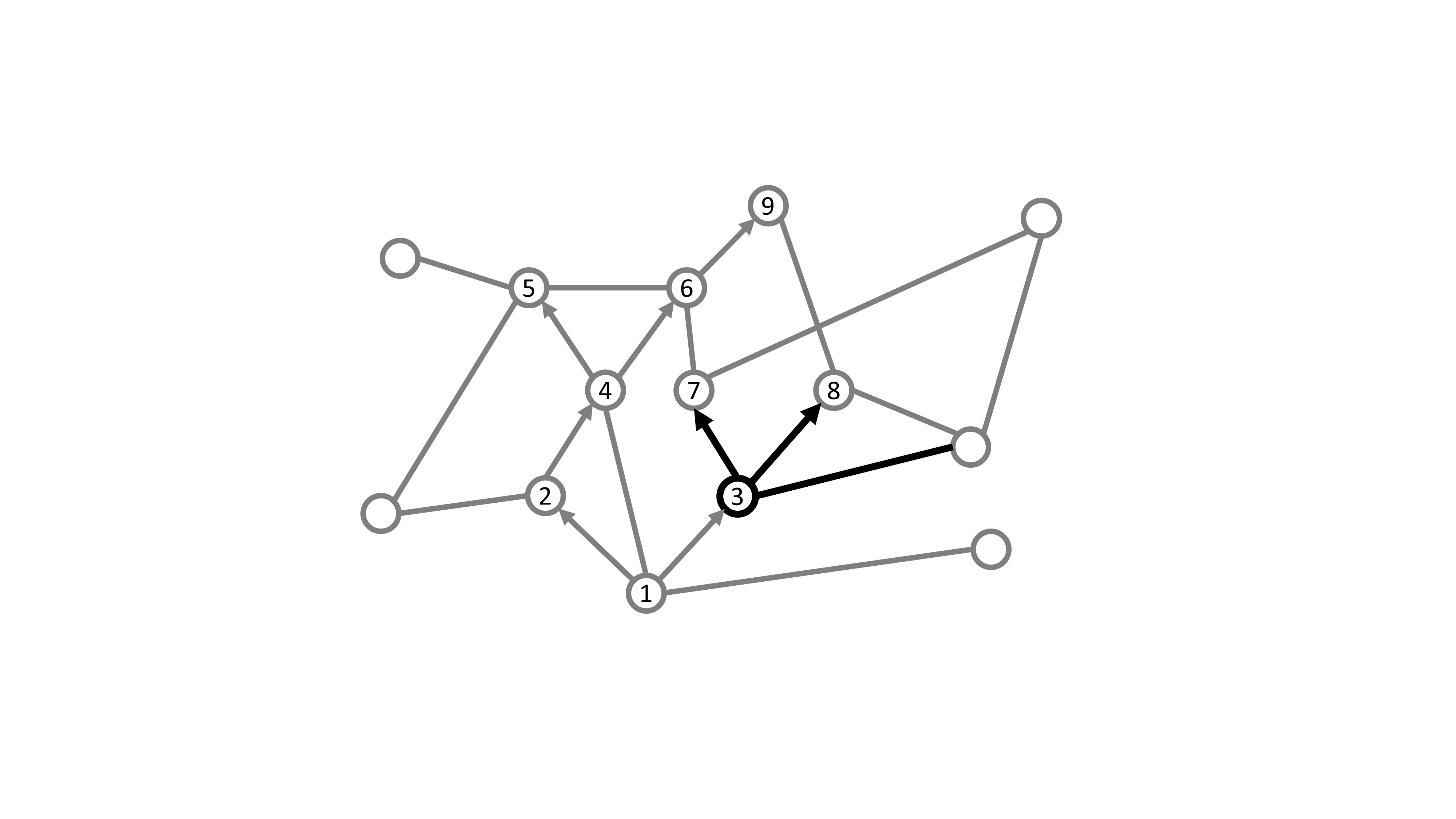

Supplement: S1 Fig — Illustration of diu, the number of unrecruited subjects connected to subject i at the moment when i is recruited. (TIF) [file pone.0185711.s002.tif]

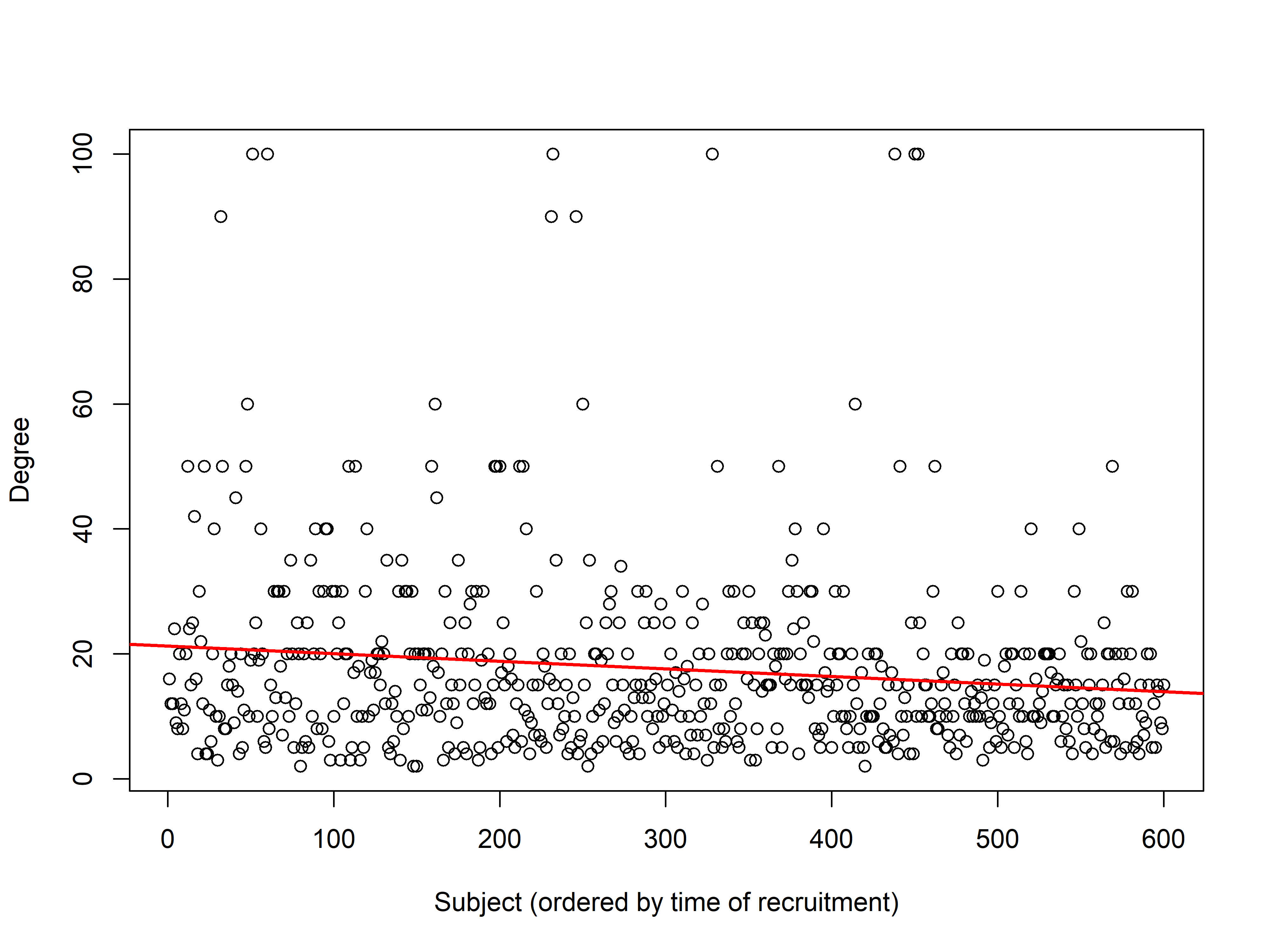

Supplement: S2 Fig — Linear regression on time-ordered network degree. (TIF) [file pone.0185711.s003.tif]
